# The Walking Egg Project: how to start a TWE centre?

**Published:** 2016-06-27

**Authors:** W Ombelet, J Goossens

**Affiliations:** Department of Obstetrics and Gynaecology, Ziekenhuizen Oost-Limburg, Schiepse Bos, 6, 3600 Genk, Belgium.; Faculty of Medicine and Life Sciences, Hasselt University, Agoralaan, 3590 Diepenbeek, Belgium.; "The Walking Egg non-profit organization".

**Keywords:** Accessible, affordable, assisted reproduction, developing countries, IUI, IVF, resource-poor countries, the Walking Egg project

## Abstract

The main objective of the Walking Egg non-profit organization is the implementation of good quality and affordable infertility centres in resource-poor countries. Three levels of assistance are suggested: A level 1 infertility clinic is a basic infertility clinic capable of offering semen analysis, hormonal assays, follicular scanning, ovulation induction and intrauterine inseminations. Level 2 infertility clinics are supposed to perform simplified IVF as well. Level 3 infertility clinics capable of offering ICSI, cryopreservation and operative endoscopy are not part of the Walking Egg Project in the initial phase. A high quality but affordable service delivery with special attention to the prevention of complications and unnecessary interventions should be the trademark of this project.

## Introduction

In the Walking Egg project we want to integrate infertility care within the concept of family planning, emphasizing that family planning centers shall be more successful if they pay attention not only in preventing unwanted pregnancies but also by promoting the chance of pregnancy in case of involuntary childlessness. Infertility treatment and family planning are in fact two sides of the same coin. Education, empowerment of women and economic prosperity are the most effective solutions to most problems related to both population growth and infertility. Reproductive autonomy, justice and equity support our efforts to make ART available and accessible worldwide subject to political stability and a basic level of medical infrastructure.

The main goal of the Walking Egg project is the implementation of good quality but low-cost infertility centres in developing countries (DC). Diagnostic and therapeutic procedures and protocols should be affordable, effective, safe and standardized.

Because resource-poor countries differ in their status of development, three levels of assistance were suggested (Sallam, 2008). A level 1 infertility clinic is a basic infertility clinic capable of offering the following services: basic infertility care including semen analysis, hormonal assays, follicular scanning, ovulation induction and IUI. In Level 2 infertility clinics IVF can be performed as well.

During many expert meetings it was decided that Level 3 infertility clinics capable of offering ICSI, cryopreservation and operative endoscopy are not part of the Walking Egg Project in the initial phase. Therefore our first target is the implementation of good quality level 2 centres.

Implementation of level 2 services entails the following activities (Sallam, 2008):

1. ***Equipping the clinics:***
  Infertility clinics in developing countries should be provided with low-cost and easy serviceable equipment taking into consideration the local problems often encountered (e.g. fluctuating voltage, frequent power cuts, unavailability of servicing facilities, irregular supply of consumables, etc...). This may require negotiations with various manufacturers to supply these tools at affordable prices, particularly if large quantities are ordered.
2. ***Training the staff:***
  This includes the training of the medical, paramedical as well as the administrative staff. Training courses should tailor to the local conditions and the possible difficulties encountered in developing countries. Table I gives an overview of the key topics covered in the training courses. Training, quality control, regular audit and systems of accreditation and registration should be implemented in order to maintain appropriate standards of care. Our objective is to organize a one or two week(s) course for all members of the team who are involved in the set-up of a pilot-centre, part of the Walking Egg Project. This training will need the support of experts in the field, who are capable to tutor the training courses at the highest level in a very short time, taking into account the experience of the trainees and the quality of facilities that can be expected in the new pilot-centres.
3. ***Educating the public:***
  This necessitates establishing contacts and working relationships with schools, community leaders, traditional healers as well as the media, producing and distributing educational materials (brochures, posters and audio-visual material) etc.
4. ***Running the services:***
  This should take into consideration staff salaries, regular purchasing of consumables, cost of equipment maintenance, cost of investigations, cost of medical interventions and the cost of medication. Special servicing contracts should be negotiated with the manufacturers. In addition, simplification of the consumables should be taken into consideration and laboratory reagents and culture media should have a long shelf life. Special prices for medication should be negotiated with the drug manufacturers and simple treatment protocols should be put into action in order to reach the best cost-effective therapies.
5. ***Documentation and registration:***
  We believe that within each pilot-centre on-line data registration of all ART activities is mandatory. Administrative staff and (para) medicals have to be aware of the importance of correct and trustable data registration. The ultimate goal is to offer all pilot-centres a similar registration programme, which should be customer-friendly with a limited but sufficient number of items (Ombelet et al., 2012). Continuous monitoring of service activities will be centralized, and provide feed-back to clinics for clinical and laboratory policy adjustments, information to couples on clinic performance, and information to society. Confidence can then be built and maintained.
6. ***Psychological and socio-cultural follow-up:***
  When implementing low-cost (accessible) infertility services in DC it is extremely important to study social, psychological, sexual, legal and ethical aspects of infertility and infertility treatment and take study findings into account when setting up gender and cultural sensitive infertility services.

**Table I:** — Detailed information about of the different steps to become a Walking Egg centre.

**Step 1**
***First contact*** with an application letter where the interested centre motivates their interest.
We need to know specifications on: The facilities Space availability? Is permanent water and electricity supply available or are there regularly discontinuances? Do you have a proper generator or water source? Instruments Detailed information about the already available equipment Work environment: Are they situated in a hospital? Is there a special program on maternal care? Do they have an endoscopic unit? Is there an IVF-centre in the neighbourhood? Informatics (IT) Does your centre have an integrated PC-system or more a stand-alone setup of PC’s? Which program do you run? Staff Members CV (educational degree) of the persons who will be working for ^t^WE-project. If possible an organogram of the gynaecological-obstetric department. Funding How will the center be organized? Will it become a private, public or private public organization?
**Step 2**
Once we have received the answers, we will send an ***NDA*** (non-disclosure agreement) and an invitation to ***subscribe to the Charter - Philosophy*** of the project. The NDA is **NOT** a contract but only a document to share all the necessary information with each other. If one agrees with the principles, both documents are signed and sent back to ^t^WE- central office.
**Step 3**
Once the NDA and the Charter are signed we will send the scenario of the project. This scenario contains all necessary information on the project and will be the basis for a tailored roll-out and implementation of the local ^t^WE-centre. At this moment a prospection visit to your centre by ^t^WE-team can be organized to speed up the implementation of the methodology.
**Step 4**
The next step is ***signing of the agreement***, which will also include a description of the tools that are delivered to your centre.
**Step 5**
Once the agreement is signed, the candidate-centre becomes a ^t^WE-centre and they can ***start*** offering the ^t^WE-treatment. The staff will receive special ***training*** by an experienced ^t^WE-team. The training will be partly held in already existing ^t^WE-centre and partly held on the location itself.
**Step 6**
Close follow-up on the medico-technical performances and the compliance with the ^t^WE Philosophy will be done by ^t^WE-staff members. In case of problems, the ^t^WE-centre can ask for assistance of the ^t^WE-staff, either on distance or on the spot. ***Regular audits*** are planned to ensure high quality standards.

### Selection of countries / pilot-centres

Decision making on infertility treatment in developing countries assumes answers to quite a few questions: How should the infertility problem be defined? How often does infertility occur? What is the income in that specific country and what to be spent on health care? How cheap should IVF be in order to be accessible to a considerable part of the population?

In this respect we believe that measurements of the (utility-measure oriented) Quality of Life over the infertile life-course in developing countries are urgently needed.

**Fig. 1:**
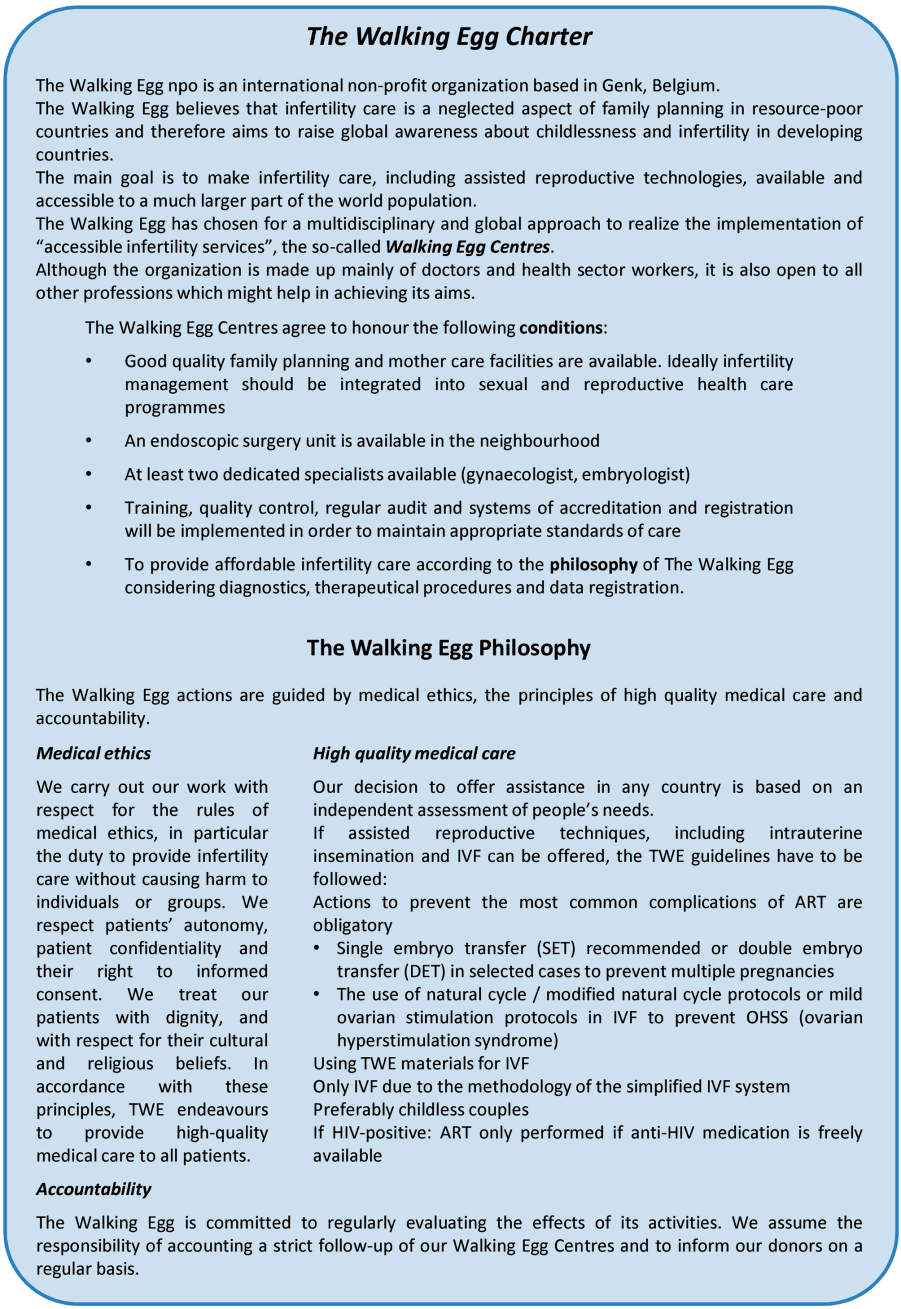
— The Charter and Philosophy of The Walking Egg Project

The selection of centres where the first pilot centres are implemented will be based on (1)available data on the resources, needs and resource gaps for infertility services on a national level, (2) percentage of GDP spent on education and health care, (3) the availability of endoscopic surgery facilities in the neighbourhood, (4) a good quality family planning unit, (5) good quality mother care facilities and (6) the availability of at least one experienced and dedicated gynaecologist and biologist (Fig. 1).

**Fig. 2:**
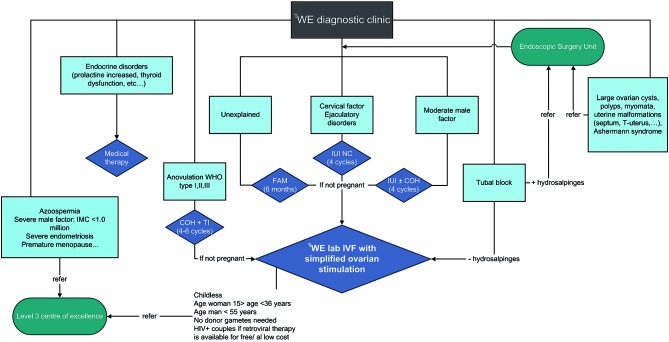
— Proposed flowchart for the ^t^WE diagnostic clinic (^t^WE = the Walking Egg, FAM = Fertility Awareness Methods, IUI = Intrauterine insemination, NC = Natural cycle, COH = Controlled Ovarian Hyperstimulation, TI = Timed Intercourse, IMC = Inseminating Motile Count).

Potential TWE centres have to agree on the philosophy and the conditions of the Walking Egg Project as described in Figure 1. The one-stop diagnostic phase with a standardized investigation of the couple at minimal costs will enhance the likelihood that infertile couples will come to the infertility centres. All the procedures of the one-day diagnostic clinic can be performed by a small team of health care providers within a short period of time in an inexpensive setting (Ombelet and Campo, 2007). A flowchart for the ^t^WE (The Walking Egg) diagnostic clinic is shown in Figure 2. Future studies are planned to assess the reproducibility of ‘one- stop infertility clinics’ in different developing countries. Minimal (low-cost) ovarian stimulation protocols to avoid multiple pregnancies and severe medical complications such as ovarian hyper- stimulation syndrome (OHSS) are mandatory, not only in an IVF setting but also in IUI (intrauterine insemination) programmes. This strategy will drop the cost for assisted reproductive technologies significantly, especially when the simplified ^t^WE IVF procedures are used (Ombelet et al., 2013; Ombelet and Campo, 2008; Van Blerkom et al., 2014).

## Application to become a TWE centre

Every centre participating in the project accepts the rules and philosophy of the concept. This implicates also that a ^t^WE-centre is by preference equipped by the ^t^WE organization. In that way, ^t^WE is also responsible for the validation, implementation and follow-up of the project.

**Fig. 3:**
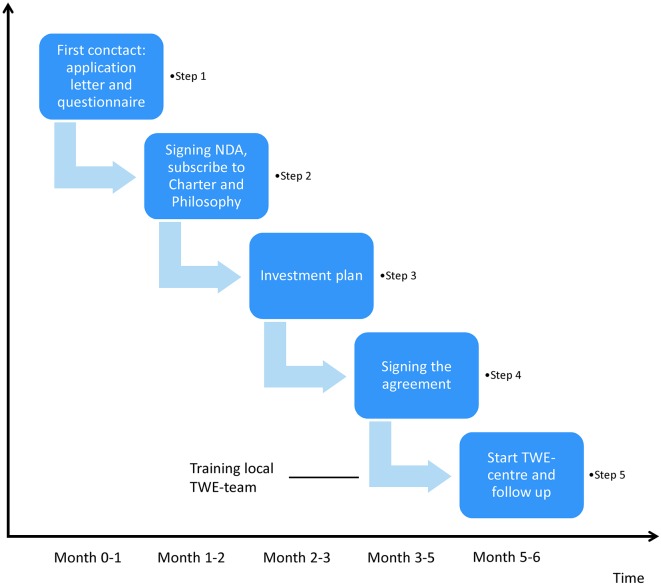
— Different steps and time frame to become a Walking Egg centre

There are 6 steps to be taken over a period of about 6 months, described in Table I and Figure 3.

## Conclusion

We believe that the Walking Egg Project can change the attitude towards childlessness and infertility care substantially, not only in the developing world, but also in our Western countries. In developing countries, family planning and mother care should be integrated in the project. A high quality but affordable service delivery with special attention to the prevention of complications and unnecessary interventions has to become the trademark of this project. Adequate training and a strict follow-up of the project are mandatory, public-private partnership may facilitate the development and implementation of Walking Egg centres and a positive relationship with and support of the local health care providers, politicians or other institutions should be stimulated.
